# Spatially uniform dynamics in equilibrium colloidal gels

**DOI:** 10.1126/sciadv.abk2360

**Published:** 2021-12-03

**Authors:** Enrico Lattuada, Debora Caprara, Roberto Piazza, Francesco Sciortino

**Affiliations:** 1 Department of Physics, Sapienza University of Rome, Piazzale Aldo Moro 2, 00185 Roma, Italy.; 2 Department of Chemistry, Materials Science, and Chemical Engineering (CMIC), Politecnico di Milano, Edificio 6, Piazza Leonardo da Vinci 32, 20133 Milano, Italy.

## Abstract

Gels of DNA nanostars, besides providing a compatible scaffold for biomedical applications, are ideal model systems for testing the physics of equilibrium colloidal gels. Here, using dynamic light scattering and photon correlation imaging (a recent technique that, by blending light scattering and imaging, provides space-resolved quantification of the dynamics), we follow the process of gel formation over 10 orders of magnitude in time in a model system of tetravalent DNA nanostars in solution, a realization of limited-valence colloids. Such a system, depending on the nanostar concentration, can form either equilibrium or phase separation gels. In stark contrast to the heterogeneity of concentration and dynamics displayed by the phase separation gel, the equilibrium gel shows absence of aging and a remarkable spatially uniform dynamics.

## Teaser

Equilibrium gels formed by the reversible association of limited-valence colloids display a remarkable spatially uniform dynamics.

## INTRODUCTION

Gels are low-packing disordered networks of interacting particles that are structurally arrested and able to support weak stresses. They are ubiquitous in nature, encompassing molecular, supramolecular, polymeric, and colloidal systems. Examples of gels include liquids that form extended open transient networks of bonds (tetrahedral in water and silica), which arrest in a permanent structure (*1*–*3*); polymer melts or solutions in which distinct chains are linked together (*4*–*6*); associating tetrahedral macromolecules (star polymers) (*7*–*9*); and colloidal solutions in which interparticle attraction (combined with particle shape) favors the formation of a porous, disordered material (*10*). Depending on the lifetime of the interparticle connections forming the network, gels can be classified as either chemical (irreversible, infinite bond lifetime) or physical (reversible, finite bond lifetime) (*4*, *5*).

Most colloidal physical gels, including several protein ones (*11*), are formed by quenching the system into a thermodynamically unstable region, separating a colloidal-rich from a colloidal-poor phase, the analogous of the gas-liquid transition in a pure fluid (*12*). For sufficiently deep quenches, the phase separation process is interrupted by a glass transition taking place in the dense phase. Then, the system remains kinetically trapped into a disordered network of rather compact clusters (*12*, *13*). Several gels, including the ones formed by arrested spinodal decomposition, are heterogeneous both in space and in time (*14*–*18*) and are characterized by aging and complex dynamics (*16*, *19*). In particular, these materials often display intermittent microscopic dynamics, consisting of relatively long quiescent stages interspersed with rapid structural rearrangements involving the whole sample (*16*, *18*, *20*). These events are caused by the relaxation of internal stresses generated during the sudden kinetic arrest (*21*) and affect the mechanical properties of the gels during their lifetime (*18*, *22*).

Polymer gels are also commonly characterized by inhomogeneous structure and dynamics, originating from the intrinsic out-of-equilibrium polymerization kinetics (*14*). Only recently, a polymer network with a high degree of homogeneity, made with tetrahedron-like macromonomers (Tetra-PEG), has been synthesized and extensively investigated (*7*, *23*–*25*). This inherently irreversible gel is produced by mixing two types of macromonomers, whose reactive groups must be accurately selected to obtain the desired homogeneity (*26*). The homogeneity has been interpreted as arising from a bond percolation phenomenon (*8*) in which the polymer units first form a homogeneous structure, which is subsequently cross-linked.

In colloidal systems, the concept of equilibrium gels has been recently developed to describe the onset of dynamic arrest at low density in the absence of phase separation (*10*, *27*–*29*). When phase separation takes no action in the gelation process, the particles are able to progressively develop a persistent network of interparticle bonds. It has been shown that limited valence is a prerequisite for shrinking and shifting the coexistence region to lower concentration and temperature values (*27*), offering a coherent thermodynamic interpretation for colloidal gelation. Limiting the number of bonds that particles can form opens up a wide window of concentrations, between the coexistence region and the glass region, where the system can be cooled down to very low *T* (substantially smaller than the attraction energy scale) without phase separation. The result is the formation of an empty-liquid state (*27*), an extensively bonded percolating network. As the system is cooled down, particles progressively create more connections until all possible bonds are formed and the system reaches its lowest energy state, forming the equilibrium gel. On further cooling, the structural properties of the gels do not change any longer and the lifetime of the network increases, mirroring the increasing bond lifetime.

Experimental evidence supporting the existence of equilibrium gels was first found in Laponite (*28*), an industrial synthetic clay composed of colloidal discotic nanoplatelets, characterized by inhomogeneous charge distribution and anisotropic interactions. Following an irreversible aggregation process, the system was observed to phase separate, but only at very low concentrations. For higher concentrations, the samples remained arrested showing no aging. Similar results were also found with other compounds (*30*–*33*), some of which were of biological interest (*34*, *35*). More recently, the flexibility offered by DNA nanotechnology (*36*), exported to soft matter (*37*), has been mastered to generate an experimental system with tunable valence and interaction strength (*38*). Experimental studies on physical gels of DNA nanostars (NSs) definitively proved the valence dependence of the phase separation region and the presence of an equilibrium gel region above the coexistence phase in which the smooth progressive slowing down of the dynamics could be explored and quantified (*38*–*41*).

Here—by exploring the gel dynamics over 10 orders of magnitude in time and resolving its spatial dependence thanks to a combination of dynamic light scattering (DLS) and photon correlation imaging (PCI), a recently introduced technique that blends light scattering and imaging (*42*)—we provide the first experimental evidence that equilibrium gels do not show any appreciable spatial or temporal dynamic heterogeneities. We do so by studying a one-component model colloidal system formed by the reversible aggregation of limited valence DNA NSs. This evidence is even more notable when compared to the behavior of the very same system obtained in conditions where gelation is induced by a rapid quench into the gas-liquid coexistence region. An equilibrium route to gelation thus appears to be a necessary condition for the formation of uniform (in space and time) gels (*8*).

## RESULTS

**Fig. 1.**
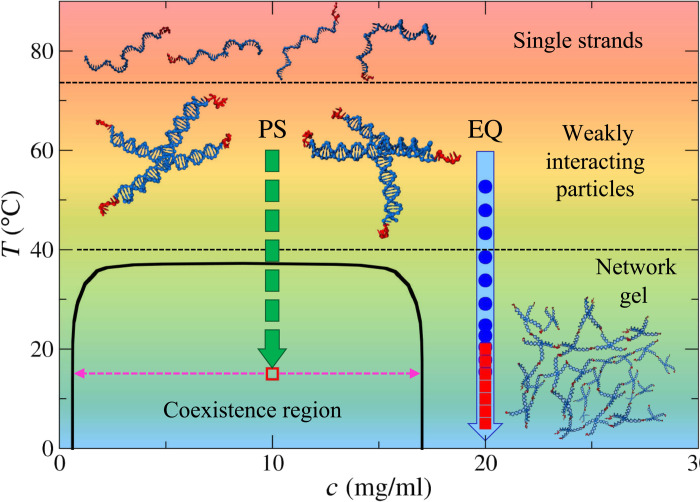
Schematic phase diagram of the system. At high *T* (> 80°C), the DNA single strands freely diffuse in the solvent. At intermediate *T* (40°C < *T* < 75°C), the strands assemble into the NS-shaped tetravalent nanoparticle. For *T* < 40°C, the particles start to interact via the sticky tips. When the DNA concentration *c* is within the range of the coexistence region (1 < *c* < 17 mg/ml), the system phase separates into dense and dilute phases for *T* < 37°C. Outside this region, the particles are able to form an equilibrium network. The coexistence line is adapted from (*38*). The arrow on the right shows the experimental path for the equilibrium gel sample EQ (*c*_EQ_ = 20 mg/ml), with the full circles and squares, respectively, indicating the temperatures where the measurements with DLS and PCI have been performed. The phase-separated sample PS, prepared at *c*_PS_ = 10 mg/ml, was instead directly quenched from 60°C to 15°C (dashed arrow), which led to rapid phase separation and kinetic slowing down.

The system that we investigate consists of tetravalent DNA NSs, which are composed of four double-stranded arms of 20 base pairs departing from a common flexible core of unpaired adenines [*M*_w_ (weight-average molecular weight) = 60,364 g/mol; for additional details, see Materials and Methods, section S1, and fig. S1]. These particles are assembled starting from four distinct single DNA strands, each composed of 49 nucleotides, containing properly designed sequences of complementary groups. Each arm terminates with a six-base long, single-stranded, self-complementary sticky sequence (CGATCG) preceded by an additional unbonded adenine, which is inserted to ease the linking between different NSs. The DNA sequences are designed so that two distinct self-assembly processes occur at well-separated temperatures (see Fig. 1). At high temperatures (*T* > 80°C), the single strands freely diffuse in the solvent and very weakly interact with each other. By slowly cooling down the suspension, the four strands start to assemble forming a well-defined NS around *T*_NS_ ≈ 75°C. Upon further reducing the temperature, around *T_b_* ≈ 40°C, the sticky ends belonging to different NSs start to hybridize forming bonds between the NSs, which become progressively longer lived on lowering the temperature. When *c* is inside the range of the coexistence region, for *T* < 37°C, the system undergoes phase separation into dense and dilute phases. The dilute phase is composed of isolated NSs, while the dense phase consists in a network of NSs, the equilibrium gel phase. The phase diagram and the *T*-dependent dynamics of the system have already been characterized (*38*, *39*, *43*, *44*).

Solutions of DNA NSs offer the unique opportunity to investigate both equilibrium and phase separation gel formation with the same system, simply by varying the particle concentration. We choose to investigate two samples at DNA concentrations *c*_PS_ = 10 mg/ml and *c*_EQ_ = 20 mg/ml, respectively, selected to be inside (sample PS) and outside (sample EQ) the range where phase separation occurs.

In addition to DLS, we use a custom-designed light scattering PCI setup to measure the local dynamics of the sample at different temperatures (i.e., different extent of bonding) with spatial resolution (see Materials and Methods for details on both setups) (*42*,*45*). The sample is placed inside a thermostatting holder, connected to a circulating bath, and is transversally illuminated by a laser sheet (λ = 638 nm) of thickness ≈60 μm. A magnified (×3.3) image, corresponding to a region of size 1.65 × 1.18 mm^2^, is formed on a complementary metal-oxide semiconductor (CMOS) sensor by the light scattered by the sample at θ = 90°, corresponding to a scattering vector *q* = (4π*n*λ^−1^) sin(θ/2) ≃ 18.6 μm^−1^ using two achromatic doublets with a pinhole placed in their common focal plane. This pinhole, besides accurately selecting the scattering wave vector (Δθ ≈ 2°), causes the image to become “speckled” because the intensity at each given point on the image plane originates from the interference of the field scattered by a finite-size region in the sample plane. Thus, the fluctuations in time of the intensity on a given speckle become a probe of the microscopic dynamics at the selected location in the sample, allowing spatial heterogeneity to be detected and quantified.

For each selected temperature, we collect a sequence of images (*N* = 3000) equally spaced in time. The time delay between two consecutive images is chosen to be proportional to the slow relaxation time at the selected temperature. The speckle pattern is then split into an array of rectangular “regions of interest” (ROIs) and the space-resolved intensity correlation between a pair of images taken at time *t* and *t* + τ evaluated as (*42*).

$$g_{2}(\tau ;r,t)=\frac{{\langle I_{p}(t)I_{p}(t+\tau )\rangle }_{\text{ROI}(r)}}{{\langle I_{p}(t)\rangle }_{\text{ROI}(r)}{\langle I_{p}(t+\tau )\rangle }_{\text{ROI}(r)}}$$ (1)

where 〈…〉_ROI(**r**)_ denotes an average of the scattered intensity over the pixels of an ROI centered in **r**. This quantity is analogous to the intensity correlation function measured by DLS but with the major advantages of (i) providing a fast ensemble averaging over all the speckles in an ROI and (ii) retaining spatial resolution. If gel aging is absent or slow compared to the duration of the experiment, the statistical accuracy can be improved by averaging over all the starting times *t* and defining *g*_2_(τ;**r**) = 〈*g*_2_(τ;**r**,*t*)〉*_t_*.

**Fig. 2.**
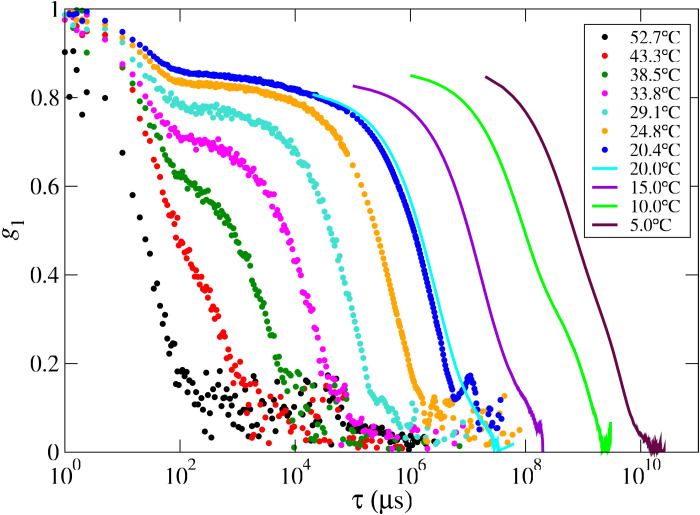
EQ sample correlation functions obtained by DLS and PCI. Correlation functions, covering 10 orders of magnitude in time, measured with DLS (dots) and PCI (lines) from the equilibrium gel sample for several selected temperatures, shown in the legend.

We first discuss the temperature dependence of the dynamics in the range 20°C ≤ *T* ≤ 55°C for sample EQ. Figure 2 shows several normalized field correlation functions $g_{1}(\tau )=\gamma^{-1}\sqrt{g_{2}(\tau )-1}$, where γ is the coherence factor of our setup (*46*), obtained by DLS. Apart from the data collected at *T* > 50°C, which can be described by a simple exponential decay function, all the correlation functions clearly display a two-step process with well-separated relaxation rates and can be fitted as the sum of a fast exponential decay plus a stretched exponential function accounting for the slower relaxation

$$g_{1}(\tau )=(1-A)e^{-\tau /\tau_{f}}+Ae^{-{\left(\tau /\tau_{s}\right)}^{\beta_{s}}}$$ (2)

where τ_f_ (τ_s_) is the fast (slow) characteristic time and *A* and β_s_ are, respectively, the amplitude and stretch exponent of the slow relaxation process, which is characterized by an average decay time

$$\langle \tau_{s}\rangle =\frac{\int_{0}^{\infty }\tau e^{-{\left(\tau /\tau_{s}\right)}^{\beta_{s}}}d\tau }{\int_{0}^{\infty }e^{-{\left(\tau /\tau_{s}\right)}^{\beta_{s}}}d\tau }=\frac{\tau_{s}}{\beta_{s}}\Gamma (\frac{1}{\beta_{s}})$$ (3)

where Γ(*x*) is the gamma function (see fig. S2 for a comparison of the data with the fit function). As already found in previous investigations, the decay time τ_f_ of the fast relaxation is weakly dependent on *T* and is not related to the gelation process but rather to the NS (collective) diffusion (*39*, *47*), and hence, it will not be discussed here. By decreasing *T*, the slow component, reflecting the network rearrangement dynamics (*39*, *47*), becomes dominant because of the gradual formation of bonds between the particles eventually leading to a fully bonded state. Concurrently, the decay of *g*_1_(τ) slows down noticeably, and the DLS measurements, requiring an overall duration 10^2^ to 10^3^ times the longest sampled delay τ to obtain sufficient statistical accuracy, become unbearably long. To quantify the dynamics below *T* ≤ 20°C, where the system is in the fully bonded equilibrium gel state, we exploit the fast ensemble averaging feature of PCI.

For a direct comparison with the DLS data, the PCI intensity correlation functions are calculated by setting the whole image as a single ROI—thus performing the averages in Eq. 1 over all the pixels of the image—and then averaging over all the initial measurement times *t*. Hence, we identify the DLS correlation function *g*_2_(τ) with the average of *g*_2_(τ;*t*) over the starting time *t*, 〈*g*_2_(τ;*t*)〉*_t_*.

The PCI field correlation functions are plotted in Fig. 2 as full lines. The very good agreement between the results obtained with the two techniques, evidenced by comparing the data obtained at *T* = 20°C (see fig. S3 for a more detailed comparison), allowed us to extend the longest measurable decay by about four orders of magnitude, amounting to an investigation of the decay time of the density fluctuations that spans over 10 decades in time.

Because of the lower limit to the accessible time scale set by the CMOS camera acquisition time, the PCI correlation functions are fitted as a plain stretched exponential, thus retaining only the slow contribution in Eq. 2 (see section S2 and fig. S2). Note that the same lower bound of the probed time scales also prevents one to get an accurate normalization of the PCI correlation functions. In Fig. 2, this has been done by matching the amplitudes of the PCI and DLS curves at *T* = 20°C.

**Fig. 3.**
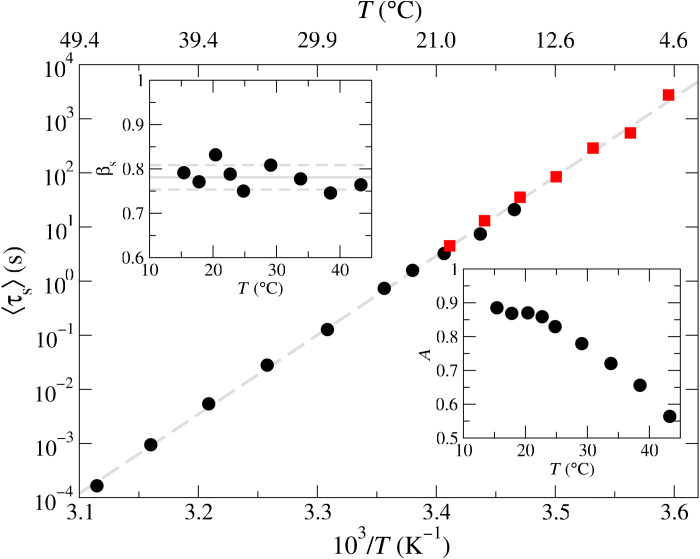
Temperature dependence of the slow relaxation. Temperature dependence of the average slow decay time 〈τ_s_〉 obtained either by DLS (dots) or by PCI (squares). The dashed line is the best fit of the experimental data using the Arrhenius equation 〈τ_s_〉 ~ exp(−Δ*H*/*RT*). The amplitude *A* of the slow relaxation contribution is shown in the lower right inset. Its stretch exponent β_s_ is plotted in the top left inset, with the lines indicating the average value ± SD.

Figure 3 shows that 〈τ_s_〉 follows an Arrhenius law in the whole temperature interval 5°C < *T* < 45°C, with its slope versus 1/*RT* (where *R* = 8.314 J/mol K is the ideal gas constant and *T* is expressed in kelvin) yielding an apparent activation energy Δ*H* = −67.0 kcal/mol. Similarly to what was found in past works on the same system (*39*, *47*), this value is roughly 1.5 times the enthalpy associated with the sticky-end hybridization Δ*H*° = −44.6 kcal/mol, calculated using the nearest-neighbors thermodynamic parameters [see (*48*)]. Notably, the stretch exponent β_s_ remains almost constant within the whole *T* range. The parameter *A*, a measure of the strength of the network, increases on cooling, reaching a plateau value when all possible bonds have been formed (*39*, *47*).

**Fig. 4.**
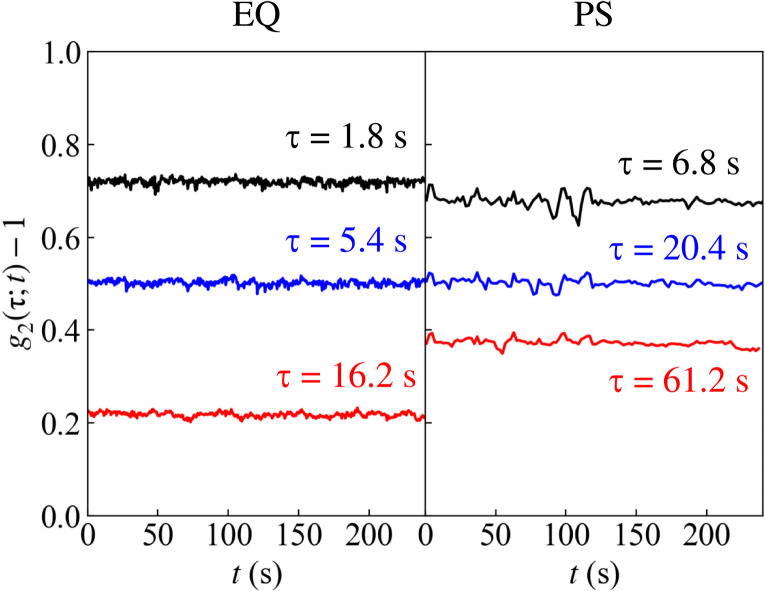
Time dependence of the intensity correlation functions of samples EQ and PS. Time (*t*) dependence of *g*_2_(τ;*t*) − 1 at fixed delay τ, indicated by the labels, for samples EQ and PS at *T* = 15°C. Note that, independently on the starting time of the measurement *t*, the value of the correlation function at delay time τ remains constant, confirming the absence of aging and the temporal homogeneity on the time scale of the measurement.

To provide evidence that samples are not aging on the time scale of the measurement, we show the starting measurement time (*t*) dependence of the intensity correlation function at fixed values of the delay τ. This can also be thought of as the value of the two-time intensity correlation function (*49*)

$$g_{2}(t_{1},t_{2})=\frac{{\langle I_{p}(t_{1})I_{p}(t_{2})\rangle }_{p}}{{\langle I_{p}(t_{1})\rangle }_{p}{\langle I_{p}(t_{2})\rangle }_{p}}$$ (4)

(where 〈…〉*_p_* denotes an average over all the pixels of the image) along the diagonal lines *t*_2_ = *t*_1_ + τ at fixed τ (see section S3 and fig. S4). Aging would manifest as a drift in *g*_2_(τ;*t*) versus *t* at constant τ. Similarly, a temporally heterogeneous, “jittery” dynamics would result in large variations of the correlation function: For instance, a strong restructuring event of the sample at the microscale would appear as a sudden drop of *g*_2_(τ;*t*) − 1 to very low values, associated with abrupt changes of the speckle pattern. These events usually repeat in time as the sample releases internal stresses [see, for instance, figures 3, 4a, and 6 of (*16*)]. Figure 4 shows *g*_2_(τ;*t*) − 1 as a function of time and at fixed values of τ, indicated by the labels. The correlation functions of both samples EQ and PS display a constant behavior over the whole experiment, attesting the absence of aging on the time scale of the measurement and temporal uniformity of the dynamics. This homogeneity allows us to improve the statistical accuracy of the PCI correlation function by averaging the measured *g*_2_(τ;*t*) over the duration of the experiment. The previous positive comparison with DLS results also allows us to count on PCI for evidencing the high degree of spatial uniformity of the local dynamics in sample EQ, in particular, when contrasted with that of sample PS.

As alluded to previously, by lowering *T* below 37°C, sample PS incurs into a phase separation heralded a few degrees above by a strong increase of the scattered intensity attesting the increase of the density fluctuations in the system on approaching the thermodynamic instability line (see section S4 and figs. S5 and S6). However, when the sample is rapidly quenched into the coexistence region, phase separation is slowed down—or even arrested—by the network formation process (*12*, *50*). To generate a phase separation gel, we rapidly quenched down sample PS from 60° to 15°C and then let the resulting gel age for a day before investigating its dynamics by PCI.

Spatial inhomogeneities in the dynamics are very effectively visualized by means of so-called “correlation maps,” two-dimensional matrices where the values of the net intensity correlation function *g*_2_(τ;**r**) − 1 on each ROI is plotted on a color scale for a fixed value either of the delay τ or of the space-averaged correlation function 〈*g*_2_(τ;**r**) − 1〉_**r**_ (namely, of the mean value *C* of this quantity over the whole map). To this aim, we subdivided the PCI images into equal ROIs by grouping together 25 × 25 adjacent pixels, corresponding to a surface area in the sample plane of scattering of 49 × 49 μm^2^, and computed the intensity correlation functions on each ROI.

**Fig. 5.**
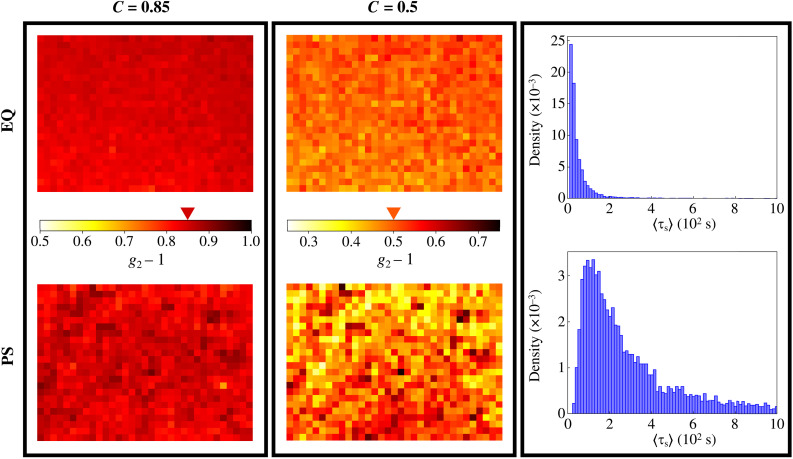
Correlation maps and distribution of slow decay times. Correlation maps at *T* = 15°C, showing the local values of *g*_2_(τ;**r**) − 1 when the spatially averaged correlation function 〈*g*_2_(τ;**r**) − 1〉_**r**_ has attained a value *C* = 0.85 (left) and *C* = 0.5 (middle). These values correspond to τ = 0.5 s and 1 s, for *C* = 0.85, and to τ = 5.4 s and 17.3 s, for *C* = 0.5, for sample EQ and PS, respectively. The scale bars mapping the color to the value of *g*_2_(τ;**r**) − 1 are shown in between the corresponding correlation maps; the triangle indicates the selected value of *C*. Panels on the right show histograms of the characteristic time 〈τ_s_〉 obtained by fitting all the computed correlation functions. Panels in the top and bottom rows refer to samples EQ and PS, respectively. Notice that here the values of the correlation functions are not rescaled, contrarily to what was done in Fig. 2 to compare the data with the DLS measurements.

Left and central panels in Fig. 5 show the correlation maps obtained at *T* = 15°C for both samples EQ (top) and PS (bottom) and for two values of the average “correlation index,” *C* = 0.85 and *C* = 0.5, respectively. Here, the color of each pixel indicates the value of *g*_2_(τ;**r**) − 1 in the imaged sample position **r**. A wide distribution of colors thus indicates a spatially heterogeneous dynamics. In contrast, a monochromatic map indicates that dynamic processes are identical in all points of the sample. The panels clearly show that, already when *C* = 0.85, the dynamics in sample EQ is much more uniform than in sample PS. The dissimilarity between the two samples becomes notable when we contrast the correlation maps for *C* = 0.5, namely, when the spatially averaged correlation function has decayed to half of its initial value. This is further highlighted by the panels on the right, which show the full distributions over all the ROIs of the characteristic slow relaxation time 〈τ_s_〉.

We also computed the “four-point” correlation function *G*_4_(Δ*r*,τ), which quantifies the spatial correlation of the local dynamics at distance Δ*r* (*42*, *51*)

$$G_{4}(\Delta r,\tau )={\langle \frac{{\langle \delta g_{2}(\tau ;t,r_{1})\delta g_{2}(\tau ;t,r_{2})\rangle }_{t}}{\sigma (\tau ;r_{1})\sigma (\tau ;r_{2})}\rangle }_{|r_{2}-r_{1}|=\Delta r}$$ (5)

where δ*g*_2_(τ;*t*,**r**) = *g*_2_(τ;*t*,**r**) – 〈*g*_2_(τ;*t*,**r**)〉*_t_* and $\sigma (\tau ;r)=\sqrt{{\langle \delta g_{2}^{2}(\tau ;t,r)\rangle }_{t}}$. We find that the spatial correlation decays to zero over one ROI (see fig. S7), contrarily to what is usually found for colloidal gels, which are known to develop long-range spatial dynamic correlations (*42*).

## DISCUSSION

In summary, by exploiting the possibility to follow two different gelation routes with the same sample, we have provided evidence that equilibrium gels are characterized—besides the absence of aging—by a spatially homogeneous dynamics. Sample EQ shows indeed a notable spatial uniformity, which has never been reported previously in colloidal gels. Space homogeneity is particularly important in biologically relevant gels, which form eye lenses (*34*), or to hydrogels, which are investigated now for biomedical applications as scaffolds, cell culture medium, and drug delivery (*52*). The behavior of sample PS in which coarsening leaves its imprints in the structure and dynamics of the gel instead strongly parallels that of phase-separated depletion colloidal gels (*16*), confirming the role of the arrested coarsening dynamics in originating a sample inhomogeneous in space and in time. In contrast, the ability to approach the gel state via a continuous sequence of thermodynamic equilibrium steps in the concentration region intermediate between phase separation and glass formation (*27*), a possibility deriving from the limited valence, appears to be the key ingredient to the formation of dynamically homogeneous colloidal gels. This work, demonstrating spatial and dynamic homogeneity, not only deepens our understanding of the behavior and properties of equilibrium gels formed by limited-valence particles but also possibly provides a framework to interpret the recently found homogeneous polymer gels (*8*) in a unified framework. It also opens a route to connect structural properties at the mesoscale and dynamic correlations in more complex colloidal systems. Candidates are the equilibrium gels formed by reversible linker-based assembly of colloidal nanoparticles (*53*).

## MATERIALS AND METHODS

### Sample preparation

DNA sequences are purchased from Integrated DNA Technologies with polyacrylamide gel electrophoresis purification. Both samples EQ and PS are prepared by initially suspending the lyophilized samples in filtered, deoxyribonuclease–free, 50 mM NaCl solution and then mixing equimolar quantities of the single DNA strands into square borosilicate glass capillaries with an inner size of 2.4 mm (Hilgenberg GmbH) to obtain a total volume of 40 μl in 250 mM NaCl aqueous solution. The samples, covered with a thin layer of silicone oil and sealed with ultraviolet-curable resin to avoid sample evaporation, are finally incubated for 20 min in an oven at 90°C and then slowly cooled down to room temperature overnight to allow for the formation of the NSs.

### DLS measurements

DLS measurements are carried out at a fixed angle θ = 90° using a custom-made setup consisting of a 633-nm He-Ne laser (17 mW, Newport Corp.) and a multi-tau digital correlator (Brookhaven Instruments) connected to an optical fiber. The sample is immersed in a water bath connected to a thermostat. For each selected temperature, the sample is thermalized for 40 min before starting the acquisition. The autocorrelation functions of the scattered intensity *g*_2_(τ) are calculated from the correlator output and converted into the field correlation functions *g*_1_(τ) using the Siegert relation (*46*).

### PCI setup

PCI measurements are carried out using a custom-made setup (a sketch of the setup is shown in fig. S8). An 8-mm uniform vertical laser line, generated using a Powell lens and a cylindrical lens in series, is focused (≈60 μm) on the sample using a second cylindrical lens. The light scattered by the sample at θ = 90° is collected by two lenses with focal lengths *f*_1_ = 75 mm and *f*_2_ = 250 mm, respectively, and an image of the interference of the scattered light is generated on the sensor of a CMOS camera (Dhyana 400D, Tucsen Photonics Co. Ltd.; sensor size, 2040 × 2048 pixels; pixel size, 6.5 × 6.5 μm^2^). The sample and the camera are placed at a distance *f*_1_ and *f*_2_ from the first and second lenses, respectively. A pinhole, which is used to select the scattering angle (Δθ ≈ 2°), is placed in the common focal plane of the two lenses.

## Supplementary Materials

### This PDF file includes:

Supplementary Text Figs. S1 to S8 References
