# Supplementary material for: Spatially uniform dynamics in equilibrium colloidal gels
Source: Sci Adv. 2021 Dec 3;7(49):eabk2360. doi: 10.1126/sciadv.abk2360 (PMC8641940; doi:10.1126/sciadv.abk2360)
Supplement: Supplementary file 1 — Supplementary Text Figs. S1 to S8 References [file sciadv.abk2360_sm.pdf]

Science Advances
AAAS

Supplementary Materials for

Spatially uniform dynamics in equilibrium colloidal gels

Enrico Lattuada, Debora Caprara, Roberto Piazza, Francesco Sciortino*

*Corresponding author. Email: francesco.sciortino@uniroma1.it

Published 3 December 2021, *Sci. Adv.* **7**, eabk2360 (2021)
DOI: 10.1126/sciadv.abk2360

This PDF file includes:

Supplementary Text
Figs. S1 to S8
References

Supplementary Text

Section S1 – DNA nanostars: sequences and hierarchical self-assembly

The selected custom-designed DNA sequences were already used in previous investigations (*38, 39*). The DNA nanostars (NSs) are composed of four arms of 20 base pairs, departing from a flexible core of 8 unpaired adenines. Each arm terminates with a single-stranded sticky-end recognition sequence of 6 oligonucleotides, which is responsible for the binding between different NSs. An additional unpaired adenine is inserted before each sticky sequence to confer more flexibility to the sticky-ends.

The specific DNA sequences of the four strands are reported below:

5'-CTACTATGGCGGGTGATAAAAACGGGAAGAGCATGCCCATCCACGATCG-3'
5'-GGATGGGCATGCTCTTCCCGAACTCAACTGCCTGGTGATACGACGATCG-3'
5'-CGTATCACCAGGCAGTTGAGAACATGCGAGGGTCCAATACCGACGATCG-3'
5'-CGGTATTGGACCCTCGCATGAATTTATCACCCGCCATAGTAGACGATCG-3',

where complementary sequences are indicated by the same color.

The different length of the complementary sequences of the arms and sticky-end is responsible for the two-step hierarchical self-assembly process. To provide an estimation of the melting temperature of the DNA strands, we use the NUPACK oligo simulator (*54*). Based on SantaLucia thermodynamic calculations, NUPACK provides the fraction of bonded base pairs of complementary DNA sequences as a function of temperature, at fixed strand and salt concentrations.

We performed the analysis for both the NS arms and the sticky-end for a salt concentration of NaCl 250 mM. For each of the strands forming the NS arms, the concentration is fixed to c = 331 µM. The resulting sticky-ends concentration is four times larger, namely c = 1325 µM.

Fig. S1 shows the fraction of unbonded base pairs for the two assembly processes. We find that the NSs assemble around $T_{NS} \simeq 78$ °C, while the binding between NSs takes place at $T_b \simeq 40$ °C.

Section S2 – Correlation functions fit

In Fig. S2 we compare the experimental field correlation functions $g_1(\tau)$ and the fit curves for some selected temperatures. For the DLS data, Eq. (2) of the main text was used, while for PCI, we kept only the slow relaxation component of the fit function:

$$g_1(\tau) = A\mathrm{e}^{-(\tau/\tau_s)^{\beta_s}}.$$

For some temperatures (i.e., 12.5 °C and 10 °C), the presence of small bubbles/impurities, which could not be removed from the sample, affected the dynamics at large delay times. The fit was then performed on the first part of the slow relaxation (not affected by the spurious component), discarding the data points at larger delays. This procedure is shown in Fig. S2 for the data obtained at 10 °C. The values of τ_s and β_s obtained are consistent with the values obtained at all the other temperatures (namely, τ_s follows the Arrhenius behavior and all β_s are similar).

Section S3 – Two-time correlation plot

The intensity correlation function described by Eq. (1) in the main text can also be restated in the equivalent form of a two-time correlation function

$$g_2(t_1, t_2, \boldsymbol{r}) = \frac{\langle I_p(t_1) I_p(t_2) \rangle_{\mathrm{ROI}(\boldsymbol{r})}}{\langle I_p(t_1) \rangle_{\mathrm{ROI}(\boldsymbol{r})} \langle I_p(t_2) \rangle_{\mathrm{ROI}(\boldsymbol{r})}}.$$

In Fig. S4, we show such correlation functions, evaluated by setting the ROI over the whole image, for both sample EQ and PS at 15 °C. In this representation, aging would show up as irregularities along the diagonal lines (i.e., same $t_2 - t_1$ but different aging time t_1). Instead, Fig. S4 shows a quite uniform pattern, consistent with a stationary dynamics on the time scale of the measurement. The same information can also be condensed by plotting the intensity correlation function at fixed $|t_2 - t_1| \equiv \tau$ as a function of time t_1. This is the information reported in Fig. 4 of the main text. In Fig. S4, we also highlight the constant-τ lines corresponding to the data shown in Fig. 4 of the main text (the same colors are used in Fig. S4 and Fig. 4).

Section S4 – Temperature quench of sample PS

Fig. S5 shows the evolution of the speckle pattern during thermalization, following a quench from 60 °C to 15 °C for the sample PS (c_{PS} = 10 mg/ml). Before the quench, at T = 60 °C (see panel a), the speckle field is very faint (the intensity grows from black to white) and rapidly varying, suggesting the system is composed of a "gas" of NSs. When, during the quench, the temperature approaches $T \approx 40$ °C (panel b), the scattered intensity suddenly increases, attesting that the density fluctuations in the system are growing larger and larger. This opalescence is similar to the one encountered on approaching a thermodynamic instability line. Upon further cooling, the sample macroscopically separates into a NS-depleted solvent on top of a NS-rich sediment, which rearranges over time. In the case of a quench temperature of 15 °C, the sample reaches a stationary configuration about one day after the initial quench. In this final stage (see Fig. S6), the dense phase in the sample shows macroscopic, low-frequency variations of the scattered intensity when compared to the speckle field of the equilibrium gel at the same temperature, attesting the spatial heterogeneity of the concentration.

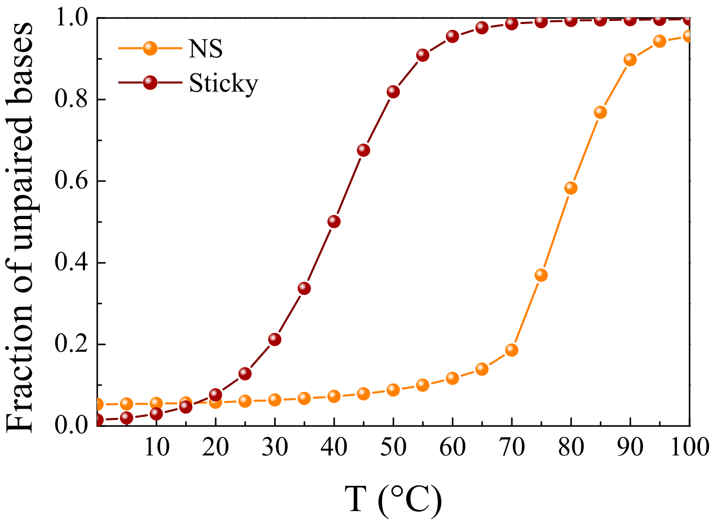

Fig. S1.

Melting profile of the DNA sequences. Melting profile of the four DNA strands forming the NS arms (orange) and of the sticky-end sequences (red), computed as described in the text using the NUPACK oligo simulator (*54*).

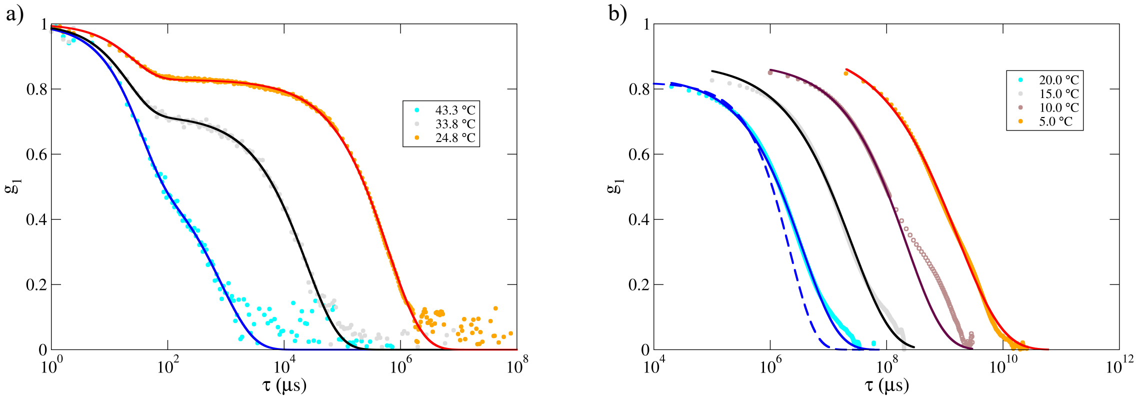

Fig. S2.

Comparison between experimental data and fit. Comparison of the experimental data (symbols) and the fit using Eq. (2) of the main text (full lines) for the field correlation functions $g_1(\tau)$ for some selected curves obtained by DLS (a) and PCI (b) at different temperatures. For the data obtained at 10 °C, displayed in panel (b), the fit was performed using only the points shown as full symbols and neglecting the open ones, to exclude the signal arising from small bubbles or large impurities, occasionally entering in the field of view. Panel (b) also shows (near the data at T = 20 °C) a simple exponential decay function (dashed line) to highlight the strength of the g_1 stretching.

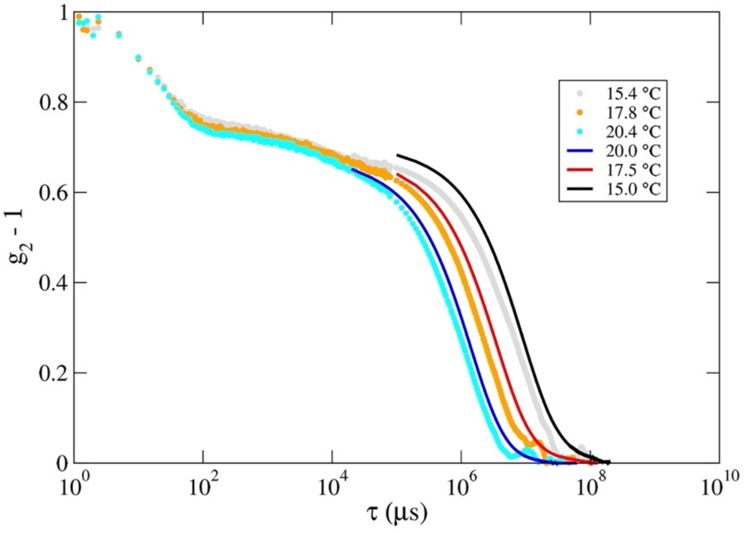

Fig. S3.

Comparison between DLS and PCI data. Comparison of the intensity correlation functions obtained by DLS (points) and PCI (lines) at the same temperatures for the sample EQ. The curves obtained by PCI are rescaled as explained in the main text.

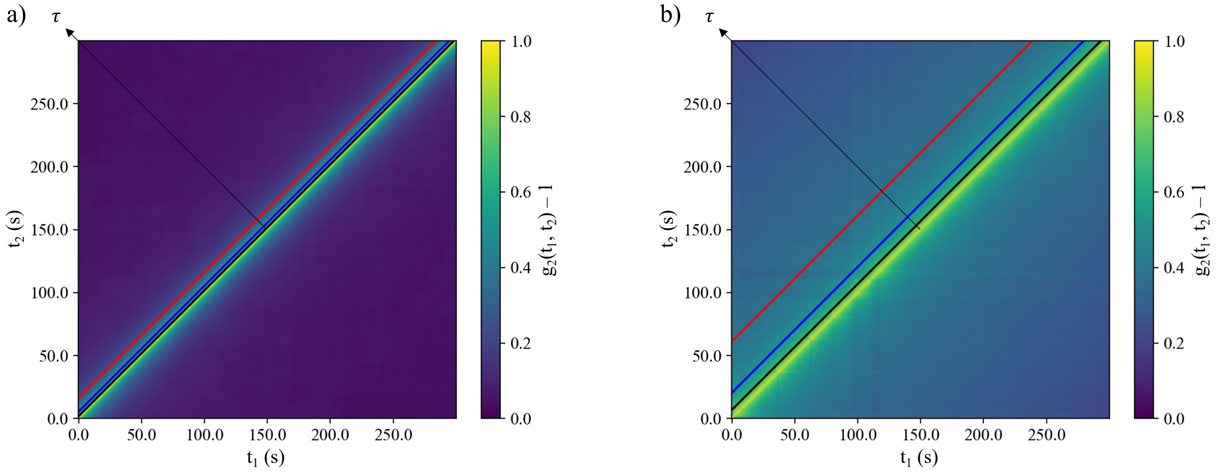

Fig. S4.

Two-time correlation plots. Two-time correlation plots, obtained as explained in the text, for the sample EQ (a) and PS (b) at 15 °C. The diagonal lines indicate the values of the delay chosen for Fig. 4 in the main text (the same colors are used in both figures).

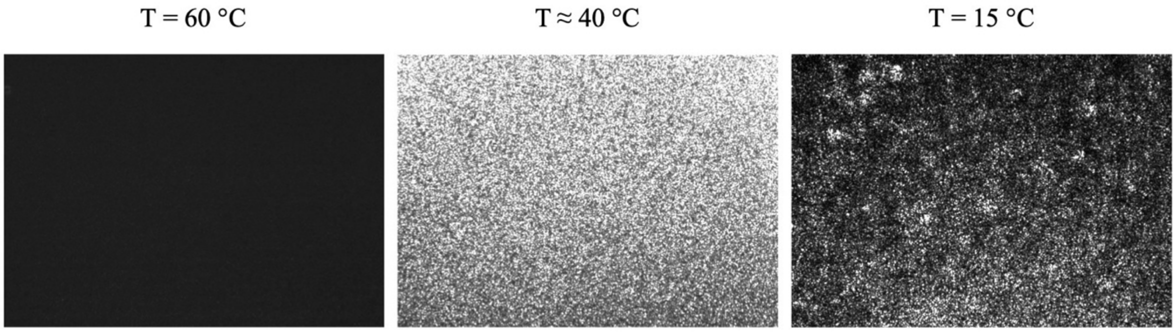

Fig. S5.

Stages of the quench of sample PS. Stages of the quench of the sample at c_{PS} = 10 mg/ml from 60 °C (left) to 15 °C (right). At $T \approx 40$ °C (center) the scattered intensity suddenly increases. The image at $T = 15$ °C was taken one day after the initial quench.

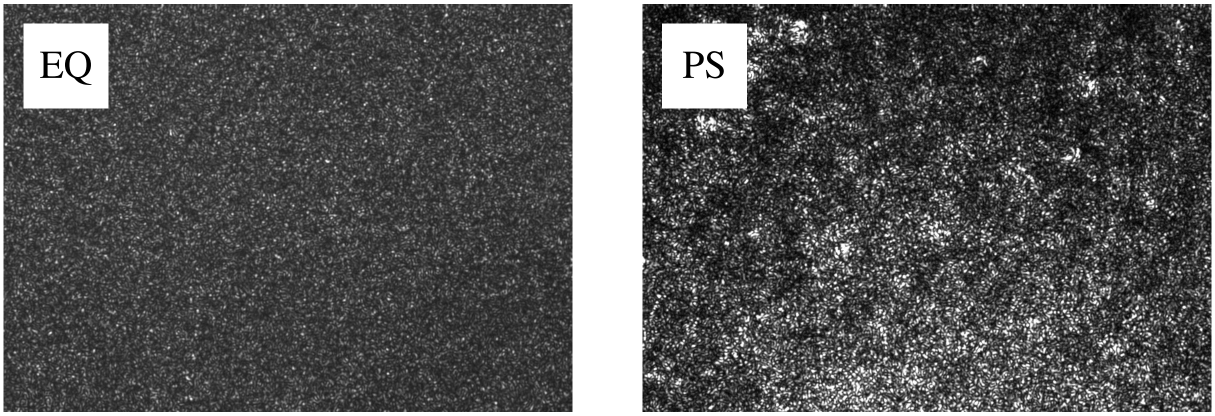

Fig. S6.

Speckle pattern comparison. Comparison between the speckle pattern of sample EQ and sample PS at $T = 15$ °C.

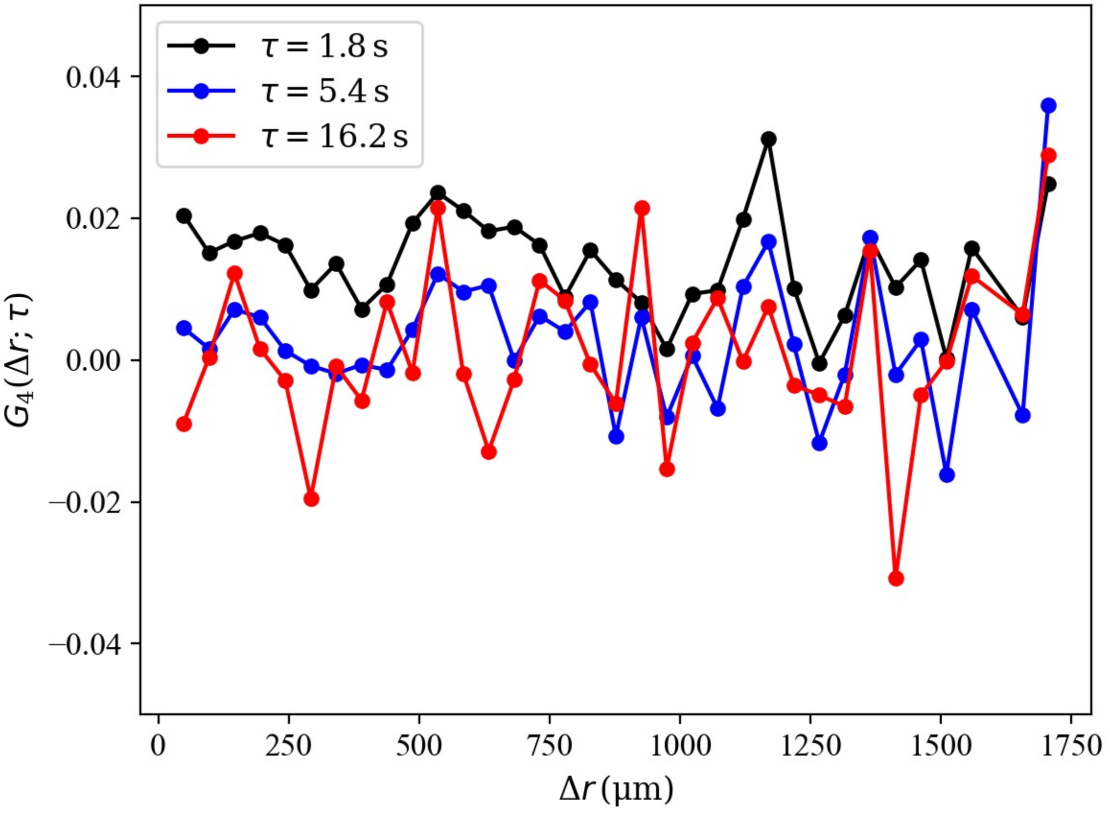

Fig. S7.

Four-point correlation function. Four-point correlation function vs the relative distance between two ROIs Δr at fixed τ: (black) 1.8 s, (blue) 5.4 s, and (red) 16.2 s (same values of Fig. 4 of the main text).

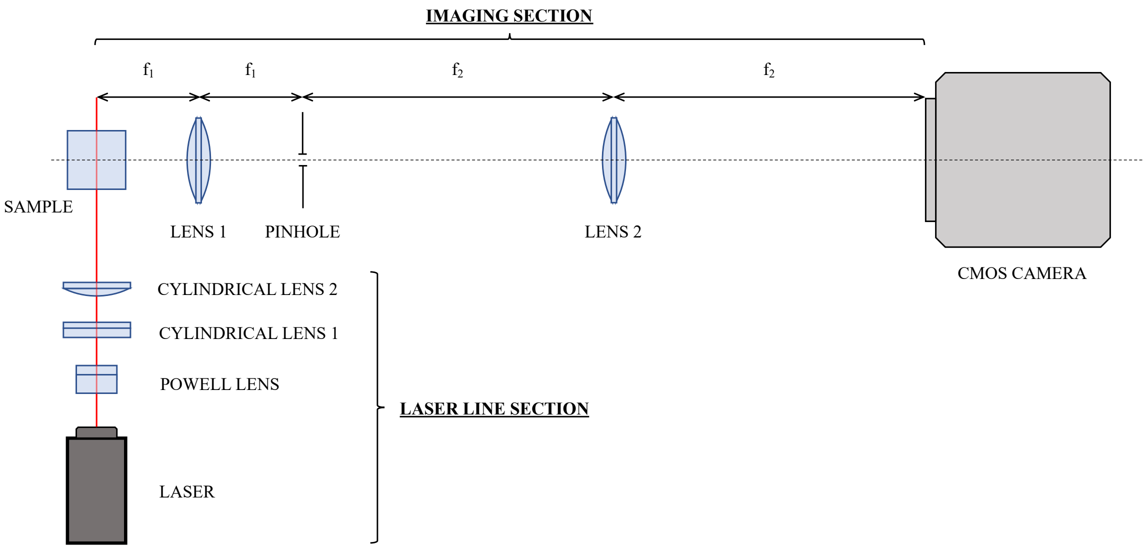

Fig. S8.

Sketch of the PCI setup. A Powell and a cylindrical lens transform the laser beam into a uniform laser sheet, which is focused on the sample by a second cylindrical lens. The light scattered by the sample at 90° is collected by two lenses and an image is formed on the sensor of a camera. A pinhole, placed in the common focal point of the two lenses, selects the scattering angle and causes the image to become “speckled”.

REFERENCES AND NOTES

1. A. Geiger, F. H. Stillinger, A. Rahman, Aspects of the percolation process for hydrogen-bond networks in water. *J. Chem. Phys.* **70**, 4185–4193 (1979).

2. H. E. Stanley, J. Teixeira, Interpretation of the unusual behavior of H_2O and D_2O at low temperatures: Tests of a percolation model. *J. Chem. Phys.* **73**, 3404–3422 (1980).

3. F. Sciortino, Gel-forming patchy colloids and network glass formers: Thermodynamic and dynamic analogies. *Eur. Phys. J. B* **64**, 505–509 (2008).

4. M. Rubinstein, R. H. Colby, *Polymer Physics* (OUP Oxford, 2003).

5. F. Tanaka, *Polymer Physics: Applications to Molecular Association and Thermoreversible Gelation* (Cambridge Univ. Press, 2011).

6. H. Kojima, T. Koga, Statistical thermodynamic theory of heat-induced gelation of aqueous methylated polyrotaxane solutions. *Macromolecules* **49**, 7015–7024 (2016).

7. T. Sakai, T. Matsunaga, Y. Yamamoto, C. Ito, R. Yoshida, S. Suzuki, N. Sasaki, M. Shibayama, U.-i. Chung, Design and fabrication of a high-strength hydrogel with ideally homogeneous network structure from tetrahedron-like macromonomers. *Macromolecules* **41**, 5379–5384 (2008).

8. X. Li, S. Nakagawa, Y. Tsuji, N. Watanabe, M. Shibayama, Polymer gel with a flexible and highly ordered three-dimensional network synthesized via bond percolation. *Sci. Adv.* **5**, eaax8647 (2019).

9. M.Shibayama, X.Li, T.Sakai, Precision polymer network science with tetra-PEG gels—A decade history and future. *Colloid Polym. Sci.* **297**, 1–12 (2019).

10. E. Zaccarelli, Colloidal gels: Equilibrium and non-equilibrium routes. *J. Phys. Condens. Matter* **19**, 323101 (2007).

11. F. Cardinaux, T. Gibaud, A. Stradner, P. Schurtenberger, Interplay between spinodal decomposition and glass formation in proteins exhibiting short-range attractions. *Phys. Rev. Lett.* **99**, 118301 (2007).

12. P. J. Lu, E. Zaccarelli, F. Ciulla, A. B. Schofield, F. Sciortino, D. A. Weitz, Gelation of particles with short-range attraction. *Nature* **453**, 499–503 (2008).

13. S. Manley, H. M. Wyss, K. Miyazaki, J. C. Conrad, V. Trappe, L. J. Kaufman, D. R. Reichman, D. A. Weitz, Glasslike arrest in spinodal decomposition as a route to colloidal gelation. *Phys. Rev. Lett.* **95**, 238302 (2005).

14. M. Shibayama, Spatial inhomogeneity and dynamic fluctuations of polymer gels. *Macromol. Chem. Phys.* **199**, 1–30 (1998).

15. T. Kogo, A. Shundo, C. Wang, K. Tanaka, Spatial heterogeneity accompanying gel formation of poly(*N*-isopropylacrylamide) aqueous solution at a temperature below cloud point. *Macromolecules* **53**, 10964–10971 (2020).

16. Z. Filiberti, R. Piazza, S. Buzzaccaro, Multiscale relaxation in aging colloidal gels: From localized plastic events to system-spanning quakes. *Phys. Rev. E* **100**, 042607 (2019).

17. L. Cipelletti, S. Manley, R. C. Ball, D. A. Weitz, Universal aging features in the restructuring of fractal colloidal gels. *Phys. Rev. Lett.* **84**, 2275–2278 (2000).

18. E. Secchi, T. Roversi, S. Buzzaccaro, L. Piazza, R. Piazza, Biopolymer gels with “physical” cross-links: Gelation kinetics, aging, heterogeneous dynamics, and macroscopic mechanical properties. *Soft Matter* **9**, 3931–3944 (2013).

19. G. Brambilla, S. Buzzaccaro, R. Piazza, L. Berthier, L. Cipelletti, Highly nonlinear dynamics in a slowly sedimenting colloidal gel. *Phys. Rev. Lett.* **106**, 118302 (2011).

20. P. Padmanabhan, R. Zia, Gravitational collapse of colloidal gels: Non-equilibrium phase separation driven by osmotic pressure. *Soft Matter* **14**, 3265–3287 (2018).

21. B. W. Mansel, M. A. K. Williams, Internal stress drives slow glassy dynamics and quake-like behaviour in ionotropic pectin gels. *Soft Matter* **11**, 7016–7023 (2015).

22. M. Bouzid, J. Colombo, L. V. Barbosa, E. Del Gado, Elastically driven intermittent microscopic dynamics in soft solids. *Nat. Commun.* **8**, 15846 (2017).

23. T. Matsunaga, T. Sakai, Y. Akagi, U.-i. Chung, M. Shibayama, Structure characterization of Tetra-PEG gel by small-angle neutron scattering. *Macromolecules* **42**, 1344–1351 (2009).

24. T. Matsunaga, T. Sakai, Y. Akagi, U.-i. Chung, M. Shibayama, SANS and SLS studies on tetra-arm PEG gels in as-prepared and swollen states. *Macromolecules* **42**, 6245–6252 (2009).

25. T. Hiroi, M. Ohl, T. Sakai, M. Shibayama, Multiscale dynamics of inhomogeneity-free polymer gels. *Macromolecules* **47**, 763–770 (2014).

26. K. Nishi, K. Fujii, Y. Katsumoto, T. Sakai, M. Shibayama, Kinetic aspect on gelation mechanism of Tetra-PEG hydrogel. *Macromolecules* **47**, 3274–3281 (2014).

27. E. Bianchi, J. Largo, P. Tartaglia, E. Zaccarelli, F. Sciortino, Phase diagram of patchy colloids: Towards empty liquids. *Phys. Rev. Lett.* **97**, 168301 (2006).

28. B. Ruzicka, E. Zaccarelli, L. Zulian, R. Angelini, M. Sztucki, A. Moussaïd, T. Narayanan, F. Sciortino, Observation of empty liquids and equilibrium gels in a colloidal clay. *Nat. Mater.* **10**, 56–60 (2011).

29. M. P. Howard, R. B. Jadrich, B. A. Lindquist, F. Khabaz, R. T. Bonnecaze, D. J. Milliron, T. M. Truskett, Structure and phase behavior of polymer-linked colloidal gels. *J. Chem. Phys.* **151**, 124901 (2019).

30. R. K. Pujala, N. Joshi, H. B. Bohidar, Spontaneous evolution of self-assembled phases from anisotropic colloidal dispersions. *Colloid Polym. Sci.* **293**, 2883–2890 (2015).

31. R. K. Pujala, H. B. Bohidar, Slow dynamics and equilibrium gelation in fractionated montmorillonite nanoplatelet dispersions. *Colloid Polym. Sci.* **297**, 1053–1065 (2019).

32. N. A. Dudukovic, C. F. Zukoski, Evidence for equilibrium gels of valence-limited particles. *Soft Matter* **10**, 7849–7856 (2014).

33. F. Zhang, F. Roosen-Runge, A. Sauter, M. Wolf, R. M. J. Jacobs, F. Schreiber, Reentrant condensation, liquid–liquid phase separation and crystallization in protein solutions induced by multivalent metal ions. *Pure Appl. Chem.* **86**, 191–202 (2014).

34. J. Cai, J. P. Townsend, T. C. Dodson, P. A. Heiney, A. M. Sweeney, Eye patches: Protein assembly of index-gradient squid lenses. *Science* **357**, 564–569 (2017).

35. J. Cai, A. M. Sweeney, The proof is in the Pidan: Generalizing proteins as patchy particles. *ACS Cent. Sci.* **4**, 840–853 (2018).

36. N. C. Seeman, *Structural DNA Nanotechnology* (Cambridge Univ. Press, 2015).

37. T. Bellini, R. Cerbino, G. Zanchetta, DNA-based soft phases, in *Liquid Crystals: Materials Design and Self-assembly*, C. Tschierske, Ed. (Springer, 2011), pp. 225–279.

38. S. Biffi, R. Cerbino, F. Bomboi, E. M. Paraboschi, R. Asselta, F. Sciortino, T. Bellini, Phase behavior and critical activated dynamics of limited-valence DNA nanostars. *Proc. Natl. Acad. Sci. U.S.A.* **110**, 15633–15637 (2013).

39. S. Biffi, R. Cerbino, G. Nava, F. Bomboi, F. Sciortino, T. Bellini, Equilibrium gels of low-valence DNA nanostars: a colloidal model for strong glass formers. *Soft Matter* **11**, 3132–3138 (2015).

40. D. T. Nguyen, O. A. Saleh, Tuning phase and aging of DNA hydrogels through molecular design. *Soft Matter* **13**, 5421–5427 (2017).

41. N. Conrad, T. Kennedy, D. K. Fygenson, O. A. Saleh, Increasing valence pushes DNA nanostar networks to the isostatic point. *Proc. Natl. Acad. Sci. U.S.A.* **116**, 7238–7243 (2019).

42. A. Duri, D. A. Sessoms, V. Trappe, L. Cipelletti, Resolving long-range spatial correlations in jammed colloidal systems using photon correlation imaging. *Phys. Rev. Lett.* **102**, 085702 (2009).

43. L. Rovigatti, F. Bomboi, F. Sciortino, Accurate phase diagram of tetravalent DNA nanostars. *J. Chem. Phys.* **140**, 154903 (2014).

44. J. Fernandez-Castanon, F. Bomboi, L. Rovigatti, M. Zanatta, A. Paciaroni, L. Comez, L. Porcar, C. J. Jafta, G. C. Fadda, T. Bellini, F. Sciortino, Small-angle neutron scattering and molecular dynamics structural study of gelling DNA nanostars. *J. Chem. Phys.* **145**, 084910 (2016).

45. R. Piazza, Optical correlation techniques for the investigation of colloidal systems, in *Colloidal Foundations of Nanoscience*, D. Berti, G. Palazzo, Eds. (Elsevier, 2014), pp. 233–266.

46. B. J. Berne, R. Pecora, *Dynamic Light Scattering: With Applications to Chemistry, Biology, and Physics* (Courier Corporation, 2000).

47. F. Bomboi, S. Biffi, R. Cerbino, T. Bellini, F. Bordi, F. Sciortino, Equilibrium gels of trivalent DNA-nanostars: Effect of the ionic strength on the dynamics. *Eur. Phys. J. E* **38**, 64 (2015).

48. J. SantaLucia Jr, D. Hicks, The thermodynamics of DNA structural motifs. *Annu. Rev. Biophys. Biomol. Struct.* **33**, 415–440 (2004).

49. A. Duri, H. Bissig, V. Trappe, L. Cipelletti, Time-resolved-correlation measurements of temporally heterogeneous dynamics. *Phys. Rev. E* **72**, 051401 (2005).

50. F. Sciortino, R. Bansil, H. E. Stanley, P. Alstrøm, Interference of phase separation and gelation: A zeroth-order kinetic model. *Phys. Rev. E* **47**, 4615–4618 (1993).

51. S. C. Glotzer, Spatially heterogeneous dynamics in liquids: Insights from simulation. *J. Non Cryst. Solids* **274**, 342–355 (2000).

52. E. Lattuada, M. Leo, D. Caprara, L. Salvatori, A. Stoppacciaro, F. Sciortino, P. Filetici, DNA-GEL, novel nanomaterial for biomedical applications and delivery of bioactive molecules. *Front. Pharm.* **11**, 01345 (2020).

53. M. N. Dominguez, M. P. Howard, J. M. Maier, S. A. Valenzuela, Z. M. Sherman, J. F. Reuther, L. C. Reimnitz, J. Kang, S. H. Cho, S. L. Gibbs, A. K. Menta, D. L. Zhuang, A. van der Stok, S. J. Kline, E. V. Anslyn, T. M. Truskett, D. J. Milliron, Assembly of linked nanocrystal colloids by reversible covalent bonds. *Chem. Mater.* **32**, 10235–10245 (2020).

54. J. N. Zadeh, C. D. Steenberg, J. S. Bois, B. R. Wolfe, M. B. Pierce, A. R. Khan, R. M. Dirks, N. A. Pierce, NUPACK: Analysis and design of nucleic acid systems. *J. Comput. Chem.* **32**, 170–173 (2011).
